# A busy BOD1-y: the diverse functions of an intracellular signaling regulatory protein family

**DOI:** 10.1042/BST20253107

**Published:** 2025-12-19

**Authors:** Thomas J. Kucharski, Martin R. Higgs, Duane A. Compton, Susanne Bechstedt

**Affiliations:** 1 Department of Anatomy and Cell Biology, McGill University, Québec, Montréal, H3A 0C7, Canada; 2 Department of Cancer and Genomic Sciences, School of Medical Sciences, College of Medicine and Health, University of Birmingham, Birmingham, B15 2TT, U.K.; 3 Department of Biochemistry and Cell Biology, Geisel School of Medicine at Dartmouth, Hanover, NH 03755, U.S.A.; 4 Centre de recherche en biologie structurale, McGill University, Québec, Montréal, H3G 0B1, Canada

**Keywords:** DNA repair, kinase, kinetochore, lysine methyltransferase, microtubule, phosphatase, replication stress

## Abstract

The biorientation-defective (BOD) protein family, comprising BOD1, BOD1L1 (BOD1-like 1), and BOD1L2, plays critical and diverse roles in fundamental cellular processes, including mitosis, DNA repair, neurological function, and metabolism. BOD1 and BOD1L2 are small proteins of less than 200 amino acids, whereas BOD1L1 contains a long C-terminal extension, totaling 3042 amino acids. BOD1 was originally identified in *Xenopus laevis* oocyte chromatin extracts. Subsequent work in mitotic human cells demonstrated that BOD1 is an outer kinetochore protein that regulates PP2A-B56 phosphatase function and consequently is vital for chromosome biorientation and segregation fidelity, hence the name. BOD1 also has important roles in neurological function and lipid metabolism as a component of the COMPASS (complex of proteins associated with SET1)–SETD1B complex. In contrast, BOD1L1 was first identified as a phosphorylated target of the ATM kinase and then highlighted in a screen for DNA replication fork components. Further work demonstrated a role for BOD1L1 in DNA double-stranded break repair, where BOD1L1 is required to recruit the protein RIF1 to damaged chromatin to enable efficient DNA repair and control sensitivity to radio/chemotherapeutics. Consistently, BOD1L1 binds known DNA damage/repair/replication proteins, including FANCD2, RIF1, and BRCA2. Loss of BOD1L1 causes catastrophic genome instability through misrepair and/or overprocessing of damaged DNA. Recently, BOD1L1 has also been shown to regulate the PP2A-B56 phosphatase at kinetochores in mitotic human cells. In contrast, little is known about BOD1L2, which is only expressed in sperm cells and precursors. In this review, we describe recent progress in understanding the functions of this protein family and discuss future avenues of research.

## BOD1 family characteristics

After the sequencing and annotation of the human genome, the biorientation-defective-1 (BOD1) group was initially designated as a family of unknown function comprising *FAM44A, FAM44B,* and *FAM44C*. The first of this trio to be functionally characterized was *FAM44B*, which was initially identified during efforts to catalog the mitotic chromatin proteome in *Xenopus* egg extracts. Four *Xenopus* proteins were identified with clear mammalian orthologues. The authors then switched to human cells and tested if knockdown of any of these orthologues resulted in a mitotic phenotype. After initial experiments revealed that human *FAM44B* is required for efficient congression of chromosomes at metaphase, it was renamed to BOD1 [1]. Accordingly, *FAM44A* was renamed BOD1L1 (BOD1-like 1, alias BOD1L), and *FAM44C* became BOD1L2.

**Figure 1:**
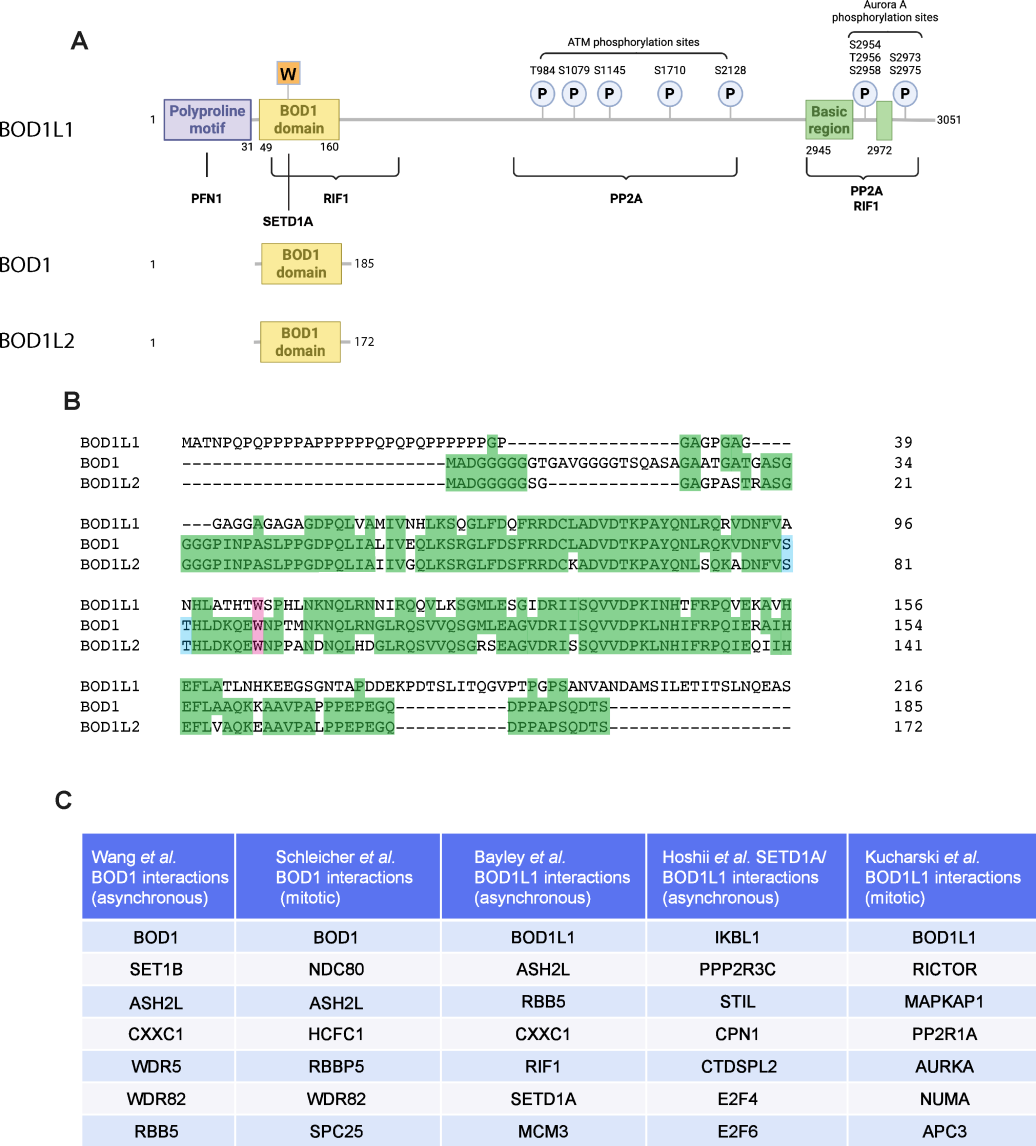
The BOD1 protein family contains three members with a high degree of similarity. (**A**) Cartoon diagram showing the relative sizes of the BOD1 family members and shared domains (not drawn to scale). The longest BOD1L1 isoform is a protein of 3051 amino acids. It contains a polyproline motif, a domain closely related to the BOD1 protein. Within the BOD1 domain, an important tryptophan residue (W104) mediates an interaction with SETD1A. Throughout the BOD1L1 sequence are several ATM kinase phosphorylation sites with unknown functions. In the C-terminus, a basic region rich in lysine and arginine amino acids serves as a nuclear localization sequence, the consensus recognition site for Aurora A kinase, and the binding surface for the PP2A B56 subunits. The RIF1 binding partner binds to the C-terminus of BOD1L1 in a manner that is yet to be fully elucidated. (**B**) Clustal Omega alignment of the N-termini of the three BOD1 family members showing the high degree of sequence identity between the family members. Identical amino acids are highlighted in green. BOD1L1 possesses an N-terminal polyproline motif that does not exist in BOD1 or BOD1L2. In contrast, BOD1 and BOD1L2 have an N-terminus rich in glycine residues. The BOD1 site that is phosphorylated by CDK1 is highlighted in blue. The tryptophan residue that binds SETD1A is highlighted in pink. (**C**) Table showing important protein–protein interactions for BOD1 and BOD1L1. Proteins highlighted in table or graphical form by Wang et al. [2], Schleicher et al. [3], Bayley et al. [4], and Kucharski et al. [5] were included. The dataset by Hoshii et al. [6] was arranged by statistical significance, and the top 5 proteins were selected. Also included were E2F4 and E2F6, as they were validated protein interactors of the BOD1L1/SETD1A complex. BOD1 is anchored to the NDC80 complex (NDC80, NUF2, SPC24, SPC25) in mitotic cells. BOD1L1 interacts with RIF1 and SETD1A to maintain genomic integrity during S-phase. BOD1L1 also interacts with E2F4/6 to control transcription. During mitosis, BOD1L1 interacts with AURKA and the PP2A phosphatase to maintain chromosome segregation fidelity. PFN1, profilin-1.

All three BOD family genes are located on separate chromosomes: *BOD1* is located on chromosome 5q35.2, *BOD1L1* is on chromosome 4p16.1, and *BOD1L2* is located on chromosome 18q21.31. *BOD1* and *BOD1L2* encode small proteins of 185 (isoform A) and 129 (isoform B) and 172 amino acids, respectively, while *BOD1L1* encodes a large protein of approximately 3051 amino acids, depending upon the isoform (Figure 1A). Since there is a high degree of DNA sequence similarity between *BOD1*, *BOD1L2,* and the 5′-end of *BOD1L1*, this suggests that these three genes arose from gene duplication events. Apart from a polyproline motif in the extreme N-terminus of the BOD1L1 protein, there is a high degree of amino acid similarity between this region of BOD1L1 and BOD1. Moreover, BOD1 and BOD1L2 are almost identical, except for a 13-amino acid insertion in the N-terminus of BOD1L2 and a slightly longer C-terminus, which aligns closely to BOD1L1 (Figure 1B). *BOD1* is very well conserved in metazoans, with identifiable orthologues present in insects. In contrast, *BOD1L1* exists only in vertebrates. Interestingly, there are almost no obvious relationships with any other proteins, with the exception of the BOD1/BOD1L1 N-terminus, which has homology with the yeast Shg1 protein [7] and the ENSA and ARPP-19 phosphatase inhibitor proteins [8]. BOD1L1 is expressed in all cell types. BOD1 is expressed in most tissue types with the exception of muscle and immune cells, and BOD1L2 appears to be expressed only in sperm cells and precursors [9,10].

Unfortunately, the protein structure of the BOD family proteins has not been elucidated. However, the BOD domain of the family members has been suggested to exist in an ordered, heat-stable conformation [8]. AlphaFold 2 structural prediction suggests that the N-terminus of BOD1L1 contains six α-helix repeats, which are important for the ability of BOD1L1 to bind to its partner SETD1A (discussed below). Targeting of the hinge between α-helices using a CRISPR-Cas9 screening approach highlighted the importance of these helices [6]. The long C-terminal tail of BOD1L1 is thought to be largely disordered, which might help enable it to bind to protein partners in multiple conformations.

## BOD1 functions in mitosis

Since BOD1 was initially discovered as a component of mitotic chromosomes, initial experiments examined mitotic progression in HeLa cells depleted of BOD1 [1]. Live cell microscopy revealed that more than 75% of these cells were unable to properly align their chromosomes or form intact metaphase plates during mitosis. Consistently, many cells underwent apoptosis. Moreover, BOD1 localizes to kinetochores, and BOD1-GFP localizes to centrosomes, the mitotic spindle, and a subset of kinetochores in cells progressing through mitosis. One obvious hypothesis from these initial findings was that BOD1 participated in the mitotic spindle checkpoint. However, cells depleted of BOD1 arrested normally in the presence of mitotic spindle poisons, ruling out that possibility. A detailed analysis of mitotic cells revealed large numbers of kinetochores that did not achieve correct bipolar spindle microtubule attachments and were instead attached to microtubules either end-on or laterally in a syntelic manner. Since unattached kinetochores were not observed, this suggests that cells lacking BOD1 suffer from hyper-stable kinetochore-microtubule (k-Mt) attachments, suggesting a loss of error-correcting ability. Since one of the main k-Mt attachment error correction pathways involves the phosphorylation and activation of the microtubule depolymerase enzyme mitotic centromere associated kinesin (MCAK, alias KIF2C) by Aurora B kinase [11–13], the authors next investigated whether loss of BOD1 from cells compromised this pathway. Although there were no obvious changes in Aurora B activity present in cells lacking BOD1, there was a substantial decrease in MCAK localization and phosphorylation at the centromere, suggesting that loss of BOD1 impairs this pathway [1].

Following their discovery of BOD1, Swedlow and colleagues continued to study the role of BOD1 in mitosis with the goal of providing mechanistic insight into the phenotypes observed in mitotic cells depleted of BOD1. They noted that BOD1 shares a number of conserved residues with the proteins ENSA and ARPP-19, which are recently characterized protein inhibitors of the PP2A-B55 phosphatase complex [14,15].

The PP2A phosphatase is composed of a catalytic (C) subunit, a structural (A) subunit, and an adaptor (B) subunit. There are two main configurations of mitotic PP2A: B55 and B56. The catalytic and structural subunits are common to both configurations, while the adaptor subunits are different. Both the catalytic and structural subunits exist as two isoforms each, of which the α isoform is more highly expressed. There are also multiple B subunits for both PP2A-B55 (αβγδ) and PP2A-B56 (αβγδε), leading to various different combinations that perform different roles within the cell [16,17]. Importantly, the roles of PP2A-B55 and PP2A-B56 are different: PP2A-B56 regulates mitotic processes such as k-MT attachments, while PP2A-B55 facilitates mitotic initiation and mitotic exit. Consistent with this notion, the B55 subunits exhibit a preference to dephosphorylate proline-directed phosphorylation sites, and the B56 subunits act on basic amino acids upstream of the phosphorylation site [18] such as those induced by the Aurora kinases.

Interestingly, while BOD1 does not bind to PP2A-B55, it does bind to the PP2A-B56 holoenzyme [8]. Similar to Ensa/Arpp-19 [14,15], BOD1 inhibits PP2A-B56 in a manner dependent on phosphorylation of threonine 95 of BOD1. This phosphorylation is mediated by CDK1 (at least *in vitro*), despite the site being a poor CDK1 consensus owing to the lack of a +1-proline residue or +3 lysine. This site is also present in BOD1L2 but not BOD1L1. Since one of the main functions of PP2A-B56 in early mitosis is the maintenance of sister chromatid cohesion through Shugoshin 1 (SGO1) [19], the authors then investigated if cohesion was affected following BOD1 depletion. Indeed, levels of the centromeric isoform B56α were increased, SGO1 levels were decreased, and BOD1-depleted cells underwent premature sister chromatid separation. They then investigated the localization and function of PLK1, which depends upon both SGO1 and PP2A-B56 for localization to centromeres [20]. Unlike other mitotic kinases, PLK1 depends on priming phosphorylation sites to bind substrates through its polo box domain [21]. Given that BOD1 functions as a PP2A inhibitor, loss of BOD1 was predicted to result in excess PP2A activity and consequently a loss of the priming phosphorylated residues required by PLK1. In agreement with this prediction, phosphorylation of an important site in CENP-U was strongly depleted, as was PLK1 kinetochore localization. Notably, cells depleted of BOD1 were hypersensitive to a chemical inhibitor of PLK1 function, suggesting a general loss of PLK1 function. In an important validation of these findings, co-depletion of BOD1 together with the PP2A adaptor subunits restored PLK1 localization and activity levels. To date, there is little structural data on the interaction between PP2A and BOD1 to generate a more complete understanding of how BOD1 regulates PP2A.

**Figure 2:**
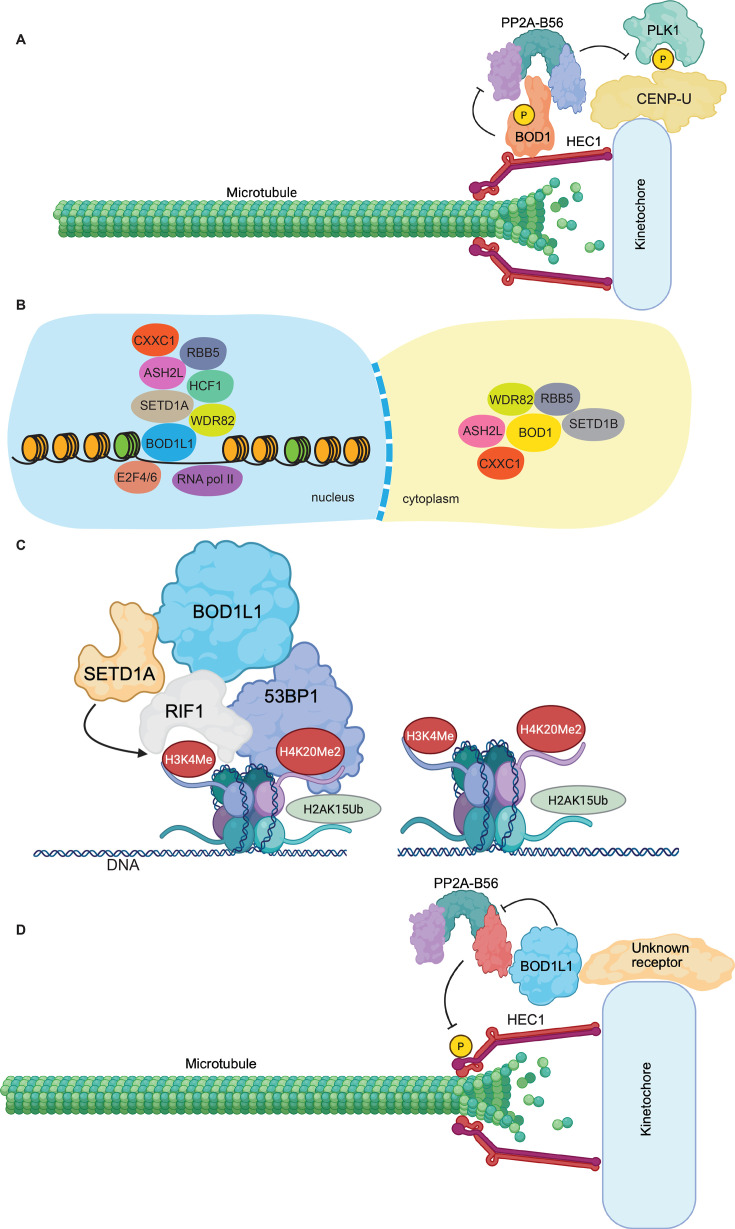
Roles of BOD1 and BOD1L1 in cells. (**A**) Cartoon diagram of how BOD1 inhibits the PP2A-B56 protein phosphatase at kinetochores during mitosis. Phosphorylated BOD1 binds to the protein HEC1 at the outer kinetochore. BOD1 simultaneously binds to and inhibits the PP2A-B56 phosphatase to increase phosphorylation levels of important kinetochore substrates such as CENP-U. Increased levels of phosphorylated CENP-U allow PLK1 to bind to the kinetochore and phosphorylate additional kinetochore substrates in a positive feedback loop. Created with Biorender.com. (**B**) Cartoon diagram depicting the nature of the nuclear BOD1L1/SETD1A complex and the cytoplasmic BOD1/SETD1B complex. The N-terminus of BOD1L1 binds DNA together with SETD1A. Also present in the BOD1L1/SETD1A complex are transcriptional regulator proteins such as E2F4/6. The BOD1L1/SETD1A complex is thus essential for proper gene transcription dynamics. In contrast, the BOD1/SETD1 complex regulates lipid metabolism in a SET domain and methylation-independent manner by correctly setting levels of genes involved in lipid metabolism. How the cytoplasmic BOD1/SETD1 complex regulates gene expression remains to be determined. (**C**) Cartoon diagram showing how BOD1L1 regulates methylation at sites of DNA repair. BOD1L1 binds simultaneously to the scaffold protein RIF1 and the SETD1A lysine methyltransferase, which methylates histone H3 on lysine 4. This methylated mark is bound by the HEAT repeats of RIF1, which together with its binding partners 53BP1 and the Shieldin complex, block resection of broken DNA by enzymes that promote DNA repair by homologous recombination. Thus, DNA repair proceeds via the non-homologous end-joining (NHEJ) pathway. Created with Biorender.com. (**D**) Cartoon diagram of how BOD1L1 binds to an unknown receptor at the kinetochore and/or on the mitotic spindle, where it binds to and inhibits the PP2A-B56 protein phosphatase using its twin basic amino acid motifs, which bind the acidic patch present on B56 adaptor proteins. Occupation of the B56 acidic patch by BOD1L1 prevents the PP2A-B56 phosphatase from binding to targets and dephosphorylating them. Therefore, BOD1L1 raises phosphorylation levels at the kinetochore on proteins such as HEC1 and KNL1. Increased phosphorylation of outer kinetochore substrates reduces binding affinity for microtubules, resulting in increased correction of errors in attachment between microtubules and kinetochores, finally resulting in increased fidelity of chromosome segregation. Created with Biorender.com.

 One of the questions arising from these findings was how BOD1 localizes to kinetochores. To determine the kinetochore receptor of BOD1, the authors performed an affinity purification-mass spectrometry (AP-MS) experiment to identify potential binding partners of BOD1 (Figure 1C) [3]. The strongest interacting protein of BOD1 was the kinetochore protein HEC1 (NDC80). HEC1 is the main load-bearing kinetochore protein that attaches to the microtubules of the mitotic spindle. It is part of the outer kinetochore and part of the KMN network consisting of KNL1-MIS12-NDC80 subcomplexes [22,23]. Since the interaction between BOD1 and HEC1 appeared robust, Swedlow and colleagues hypothesized that HEC1 might be the kinetochore receptor for BOD1, which proved to be the case. Thus, a picture emerges of BOD1 function during mitosis. BOD1 localizes to kinetochores from prometaphase to anaphase by binding to the HEC1 complex, where it simultaneously binds to and inhibits the PP2A-B56 complex, allowing phosphorylation of kinetochore proteins by mitotic kinases including Aurora A, Aurora B, PLK1, MPS1, and CDK1-Cyclin A2/B1 which are required to enable the mitotic checkpoint complex and k-MT error correction [24–30] (Figure 2A).

Although HEC1 was one of the few proteins meeting criteria for statistical significance, various other proteins were detected in this AP-MS screen, including PP2A-B56 phosphatase components [3]. Moreover, BOD1 depletion negatively affects the phosphorylation of various kinetochore proteins, including KNL1, HEC1, and CENP-U (alias PBIP1), either through loss of PP2A-B56 inhibition or indirectly by consequently affecting PP1 phosphatase or PLK1 localization to the kinetochore [3]. Thus, many of these potential interacting factors identified in the AP-MS screen might be important for BOD1 protein biology and warrant future investigation. Moreover, structural studies may also shed light on how BOD1 carries out both its HEC1 binding and phosphatase inhibition functions during mitosis.

## BOD1 functions in the nervous system

The first hint that BOD1 might have functions outside of mitosis came from a study which sought to determine if somatic copy number alterations play a role in neuropsychiatric disorders. Somatic deletions in *BOD1* were detected in individuals with schizophrenia [31]. Whole genome sequencing data from the blood, prefrontal cortex, and cerebellum of an individual with schizophrenia was used to identify copy number variations (CNVs) and genetic variations. Several germline duplications and deletions were discovered, including four that were in the genes *protein phosphatase-2 regulatory subunit B*, *gamma (PPP2R5C*) (heterozygous), *anillin, actin binding protein* (homozygous), *MYC associated factor X* (heterozygous), and *insulin-like growth factor 1 receptor* (heterozygous). Further investigation revealed 10 deletions in the brain of the same individual. Of these, different-sized deletions were found in *BOD1* in cells from the prefrontal cortex (1303 bp) compared with the cerebellum (908 bp). The deletion detected in cells from the prefrontal cortex occurred only in non-pyramidal and white matter cells, but not in pyramidal cells. Although the study was not designed to infer the cause of schizophrenia, it demonstrated that cell lineage-specific CNVs of *BOD1* do occur in the brain. These variations may be associated with neurological diseases, and a detailed investigation of the role of the genes with detected variations in brain function is warranted. Furthermore, the deletion in *PPP2R5C* is also potentially interesting given the ability of BOD1 to regulate PP2A function in mitotic cells [8], suggesting a potential role for the BOD1-PP2A pathway in neurological function.

A substantial amount of additional evidence has now been uncovered demonstrating a role for BOD1 in diverse aspects of brain function. An independent study then identified a stop mutation in *BOD1* using linkage analysis in a consanguineous family [32]. This family had four affected individuals characterized by intellectual deficiency with a pattern of autosomal recessive inheritance. Subsequent sequencing revealed a homozygous point mutation in the second exon of *BOD1* (NM_138369.2:c.334C>T; p.R112X) in these individuals. Despite not having morphological brain abnormalities, all four individuals displayed low to moderate intellectual deficiency. Interestingly, fibroblasts isolated from these individuals did not display the spindle defects and unaligned chromosomes previously observed in cancer cells depleted of BOD1. Instead, *BOD1-*mutated cells transited through mitosis more rapidly than controls. Nonetheless, these *BOD1-*mutated cells did exhibit increased levels of PP2A-B56 at kinetochores and reduced levels of PLK1. It is possible that these *BOD1-*mutated cells somehow compensate for this mutation and subsequently increase PP2A activity to promote normal mitotic progression *via* another factor (e.g. PLK1). To test this hypothesis, the authors examined the sensitivity of *BOD1-*mutated cells to PLK1 inhibition and demonstrated that these cells were far less sensitive to PLK1 inhibition, suggesting that they have either increased or bypassed the need for PLK1 function in mitosis. Moreover, *BOD1-*deficient cells display increased levels of PLK1 at centrosomes. Given the lack of a mitotic phenotype in BOD1-deficient cells, the authors speculated that the intellectual deficiency might be caused by a non-mitotic function of BOD1. To investigate this, they examined the localization of BOD1-GFP in murine corticoneuronal cells and unexpectedly demonstrated that BOD1-GFP localizes to synapses, suggesting that BOD1 might play a role in synaptic signaling. Accordingly, *Drosophila melanogaster* with reduced *BOD1* expression has significant learning defects, and the fly larvae exhibit defects in synapse branching at neuromuscular junctions.

In 2020, a second family with inherited intellectual disability was identified [33], in which whole exome sequencing revealed a homozygous nonsense-causing mutation in *BOD1* (NM_138369:c.451C>T; p.R151*). While the individuals from the first family (described above) were generally healthy except for primary or secondary amenorrhea, the affected individual in this second family displayed mild dysmorphic features, moderate hearing impairment, short stature, and endocrine dysfunction. Thus, it is possible that BOD1 might participate in other contexts besides mitosis and cognitive functioning.

Further evidence supporting a role for BOD1 in brain function arose from a study comparing twins with bipolar disorder (but differential response to lithium treatment) to their unaffected family member [34]. Interestingly, the individual who responded poorly to lithium therapy had mutations in six genes that might plausibly affect neurological functions. The mutated genes include *BOD1,* as well as *neurofibromin type 1*, *Golgi-associated gamma adaptin ear-containing ARF-binding protein 3* (*GGA3*), *disrupted in schizophrenia 1* (*DISC1*), *neuromedin U receptor 2* (*NMUR2*), and *huntingtin-interacting protein 1-related*. Only three of these mutations (*GGA3, NMUR2,* and *DISC1*) were observed in the twin who responded well to lithium. Mutations in *BOD1* might therefore affect the response of patients to treatment, which may plausibly be linked to its roles in schizophrenia and intellectual deficiency, or in other pathways.

****Additional insights into the role of *BOD1* in brain function came from a recent study on the function of a circuit between the cerebellar lobe IV/V and fastigial nucleus in a mouse model of ataxia [35]. Interestingly, *BOD1* and *RPS3* were the only two genes identified by transcription profiling of this pathway, and *BOD1* is consistently highly expressed in brain tissue, particularly cerebellar Purkinje cells. To determine whether *BOD1* loss could induce ataxia, a conditional knockout model was generated in which *BOD1* could be selectively depleted from cerebellar Purkinje cells. Loss of *BOD1* expression in this setting resulted in ataxia but had no impact on the size of the cerebellum, brain mass, gross morphology, Purkinje cell density, or electromyography. However, *BOD1* deletion did result in decreased Purkinje cell excitability, which was likely due to a decrease in the number of mature dendritic spines in these cells.

****Further evidence of a role for BOD1 in brain function comes from a study that investigated the role of BOD1 in the context of autism spectrum disorder (ASD). Dysfunction of parvalbumin-positive (PV+) interneurons has been implicated in ASD. Knockout of *BOD1* in PV+ interneurons led to an ASD-like phenotype in mice. Consistent with the Purkinje cells mentioned above, *BOD1* knockout led to hypoactivity and an increase in the resting membrane potential of PV+ interneurons. However, *BOD1* knockout also causes hyperactivity of calcium/calmodulin-dependent protein kinase IIα neurons in the prefrontal cortex. Expression of *BOD1* in *BOD1* knockout mice rescued the phenotype of *BOD1* knockout, demonstrating that *BOD1* is an important gene for neurological functioning [36]. However, the mechanism of how BOD1 contributes to neuronal function remains unknown.

## The role of BOD1 in transcription

The complex of proteins associated with SET1 (COMPASS) is a highly conserved protein complex containing either the SETD1A, SETD1B, or KMT2A-D histone methyltransferase proteins and additional subunits. Although closely related, SETD1A and SETD1B have distinct functions, supported by multiple pieces of evidence in mouse and cellular models [2,37–41]. Although both enzymes encode a conserved lysine methyltransferase function, they also have non-catalytic roles.

To define the function of the COMPASS complex, AP-MS screening was performed, which identified interactions between BOD1 and the COMPASS complex [42]. Additional studies reinforced and refined these conclusions, revealing interactions between SETD1B and BOD1 in the cytoplasm (Figure 2B). Importantly, BOD1 and SETD1B mutually stabilize each other, as loss of one subunit results in rapid degradation of the other. RNA-seq analysis was then performed in cells depleted of SETD1B or BOD1 to determine the function of this complex. Depletion of either protein resulted in extensive gene expression changes, although only a ~50% overlap was observed between SETD1B or BOD1 single depletions. Several of the dysregulated genes are involved in lipid metabolism, including *ADIPOR1, PRKAR2A, COX7C, SDC4,* and *COQ7* [2]. In agreement, the SETD1B/BOD1 complex also interacts with the mitochondrial protein HADHA/B. To determine if SETD1B/BOD1 participates in lipid signaling, BOD1/SETD1B-deficient cells were depleted of ADIPOR1 using CRISPR/Cas9, and changes in gene expression were determined. Critically, most genes regulated by SETD1B/BOD1 were rescued in AdipoR1 knockout cells*,* and cells were protected from death induced by loss of SETD1B [2]. Taken together, these data indicate that BOD1 plays a critical role in regulating gene expression and lipid metabolism, although how this occurs and how non-methylation-dependent functions of SETD1B are involved remains to be determined.

Further evidence of BOD1 functions in transcription comes from the study mentioned above in which the contribution of BOD1 to neuronal function was investigated. The authors performed transcriptional profiling in *BOD1* knockout neurons, which revealed significant changes in gene expression, including changes in several genes related to dendritic spine morphogenesis, including *MALAT1*, *FUS*, *NHRNPR, SYNE1*, *EEA1*, *CNIH2,* and *STRN* [35]. Thus, BOD1 contributes to transcription of genes involved in lipid metabolism and neuronal function, although how exactly it does so remains unknown.

## BOD1L1 functions in transcription

The first suggestion that BOD1L1 might have functions in regulating transcription came from the same study that identified an association that occurs in the cytoplasm between BOD1 and the SETD1B complex. They also identified an interaction that takes place in the cell nucleus between BOD1L1 and the SETD1A methyltransferase protein [2] (Figure 2B). SETD1A is a critical factor for maintaining correct transcription of genes, as it is an important binding partner of cyclin K, which is a cofactor of CDK proteins that control the transcriptional activity of RNA polymerase II. In this context, the enzymatic activity of SETD1A is not required [40,43]. However, SETD1A enzymatic activity is required for the proper transcription of a subset of genes in the context of embryonic stem cell (ESC) differentiation [44].

Important insights into the relationship between BOD1L1 and SETD1A were obtained from a study in leukemic cells where BOD1L1 knockout potently induced apoptosis. Interestingly, expression of BOD1L1 is essential for the stability of SETD1A, as levels of SETD1A are strongly reduced in BOD1L1 knockout cells. Transcriptional profiling was performed in BOD1L1 knockout cells, which revealed that the expression of 590 genes was down-regulated, of which several participate in the Fanconi anemia pathway. Importantly, SETD1A knockout cells and BOD1L1 knockout cells displayed similar transcriptional profiles. BOD1L1 proved essential for the correct distribution of SETD1A on chromatin at transcriptional start sites. The authors discovered that SETD1A binds to a conserved tryptophan present in the N-terminal Shg1 domain of BOD1L1, BOD1, and BOD1L2 (Figure 1A and B). The tryptophan residue in BOD1L1 is essential for the recruitment of SETD1A to chromatin. It was not tested whether the tryptophan residue in BOD1 and BOD1L2 performs a similar role. Consistently, the Shg1 domain of BOD1L1 is required for leukemic cell viability. The tryptophan residue interacts with the SETD1A FLOS domain, which is important for its non-enzymatic functions in promoting transcription [6].

Further insights into the functions of BOD1L1 in transcription come from a recent preprint showing that BOD1L1 is essential for correct expression of genes that promote DNA repair in ESCs. Consistent with the study by Hoshii and colleagues [6], they found that the N-terminus of BOD1L1 interacts with SETD1A, and that it is a highly conserved α-helix within SETD1A that interacts with BOD1L1. Notably, in contrast to the study by Hoshii and colleagues, which found no difference in histone methylation levels [6], homozygous or heterozygous knockout of BOD1L1 in ESC results in a dose-dependent increase in the levels of histone H3 methylation on lysine 4 in gene promoter-proximal regions, indicating that BOD1L1 functions to restrain SETD1A catalytic activity. However, despite the increase in histone methylation, BOD1L1 knockout resulted in a decrease in expression of genes involved in DNA repair, which the authors suggest is responsible for the decrease in cell viability following knockout of BOD1L1 [45].

## BOD1L1 functions in DNA repair

Multiple studies have demonstrated an important role for BOD1L1 in DNA repair. The first cellular characterization of BOD1L arose during studies identifying proteins involved in DNA replication fork repair using isolation of proteins on nascent DNA (iPOND) coupled with mass spectrometry. Besides known DNA replication components, this screen also identified BOD1L1 present at DNA replication forks. This was confirmed using proximity ligation assays in 5-ethynyl-2′-deoxyuridine (EdU)-labelled cells and by co-immunoprecipitation between BOD1L1 and the MCM2 and MCM7 components of the DNA replication fork helicase.

Consistent with the notion that BOD1L1 participates in repair of stalled replication forks, cells depleted of BOD1L1 were exquisitely hypersensitive to agents that cause fork-stalling inter-strand DNA crosslinks. In line with a role for BOD1L1 in resolving replication stress caused by unrepaired crosslinks, the numbers of micronuclei, ultrafine anaphase bridges, and 53BP1 foci in the G1 phase were all substantially elevated in cells depleted of BOD1L1, as were levels of phosphorylated replication protein A (RPA) and γH2AX. Moreover, these cells also showed substantial levels of damage to metaphase chromosomes after exposure to cross-linking agents. These findings suggested a link between BOD1L function and the Fanconi anemia pathway of cross-link repair. Indeed, co-depletion of FANCA and BOD1L1 revealed a clear functional overlap between these two factors. However, none of these phenotypes were observed in cells lacking BOD1, reinforcing the notion that these two related proteins are functionally separable.

 To determine the role of BOD1L1 at DNA replication forks, single molecular labeling of replication forks (DNA fibers) was employed. This revealed that cells depleted of BOD1L1 showed substantially higher levels of replication fork asymmetry, suggesting that BOD1L1 promotes DNA replication fork progression. However, this phenotype was not caused by an effect on ATR-CHK1 signaling since BOD1L1-depleted cells have normal levels of CHK1 phosphorylation and activity in the presence of DNA replication stress. In ATR-deficient cells, uncontrolled origin of replication firing can result in exhaustion of the limited pool of RPA, which is an essential factor for the protection of single-stranded DNA. This can be rescued by overexpressing RPA [46]. However, in BOD1L1-deficient cells, which exhibit significantly higher levels of RPA loading and increased ssDNA present, expression of RPA had no impact, suggesting that ATR and BOD1L1 have different functions during DNA replication stress.

 Loss of Fanconi anemia pathway components, as well as the tumor suppressors BRCA1 and BRCA2, results in hyperactivity of nucleases that resect stalled DNA replication forks [47,48]. Crucially, BOD1L1 also acts to prevent excessive resection of DNA breaks, and cells lacking BOD1L1 exhibited significant fork degradation (probably over kilobases of DNA). Moreover, BOD1L1 functions alongside BRCA1 and BRCA2 in this pathway and exists within the same physical protein complex as both BRCA2 and FANCD2. Mechanistically, this was due to an inability of BOD1L1-depleted cells to recruit the replication fork stabilizing factor RAD51. This occurs specifically in response to DNA replication stress and not in response to DNA breaks induced by ionizing radiation. Thus, in cells lacking BOD1L1, a failure to recruit RAD51 causes hyperactivity of nucleases at stalled DNA replication forks, resulting in severe genomic instability.

 In a follow-up study, the observation that BOD1L1 participates in DNA replication fork protection was combined with its role in the COMPASS complex together with SETD1A [2,42]. As previously mentioned, the N-terminus of BOD1L1 displays some sequence homology to the yeast Shg1 COMPASS complex protein. Unsurprisingly, this is the only region of BOD1L1 that interacts with SETD1A, as well as being functionally important in leukemic cells [6]. Critically, cells depleted of SETD1A alone or in combination with BOD1L1 were hypersensitive to DNA cross-linking agents, suggesting that they function within the same pathway. However, cells depleted of SETD1B or BOD1 were not, further reinforcing the notion that BOD1L1/SETD1A and BOD1/SETD1B are distinct complexes [7].

 Like BOD1L1, SETD1A was required to suppress hyper-resection of stalled DNA replication forks by nucleases by stabilizing RAD51 at sites of damage [7]. Furthermore, the lysine methyltransferase activity of SETD1A is required for this function, although its role in interacting with RNA polymerase II was not. Moreover, BOD1L1 and SETD1A were required for efficient H3K4 methylation at DNA replication forks as well as across the genome. This was independently verified through ectopic expression of an H3 mutant that cannot be methylated (H3K4A), which resulted in similar cellular defects as loss of BOD1L1/SETD1A. Finally, this pathway was also dependent on the Fanconi anemia protein and histone chaperone FANCD2, which binds methylated H3K4 and acts to mobilize H3 at stalled replication forks, allowing RAD51 access. Thus, BOD1L1 functions in a pathway where it binds to and is required for SETD1A methyltransferase activity at stalled replication forks, recruiting FANCD2 and mobilizing histones to stabilize RAD51 and prevent excessive replication fork degradation [7].

 Further details into how BOD1L1 functions during DNA replication came from a study examining the nuclear functions of profilin-1 (PFN1). This protein binds to actin and to proteins containing polyproline motifs to regulate their function [49]. For example, the nucleosome remodeling protein SNF2H contains a polyproline motif and binds to PFN1 at replication forks under unstressed conditions. This promotes the initiation and progression of DNA replication. However, under conditions of replication stress, PFN1 binding to SNFH2 increases fork stalling and enhances fork resection. In this context, PFN1 also binds to the polyproline motif in the N-terminus of BOD1L1 and reduces its fork protection activity, adding a layer of negative regulation to BOD1L1 and providing a function for the polyproline motif present in BOD1L1 (but absent from BOD1 and BOD1L2) [50].

Since BOD1L1 is also phosphorylated following ionizing radiation [51], a more recent study examined the role of BOD1L1 in double-strand break (DSB) repair. A co-immunoprecipitation-mass spectrometry approach identified multiple interacting proteins of BOD1L1, including the COMPASS components SETD1A, ASH2L, CXXC1, and RBBP5, and the DNA repair protein RIF1 (Figure 1C). RIF1 promotes DSB repair via the non-homologous end joining (NHEJ) pathway and suppresses homologous recombination [52–56]. Both the N-terminal Shg1 homology (SHG1H) domain and the hitherto-uncharacterized C-terminus of BOD1L1 mediate this interaction with RIF1 (Figure 1A). Unsurprisingly, SETD1A also interacts with RIF1, and both BOD1L1 and SETD1A promote accumulation of RIF1 to DSB sites, even though neither BOD1L1 nor SETD1A forms damage-induced ‘foci’ at DSBs in the manner of other DNA repair factors such as 53BP1 [57,58]. Nevertheless, both proteins are present at DSBs when analyzed by chromatin immunoprecipitation at *Fok1-*induced DSBs.

Given the essential contributions of RIF1 to NHEJ-mediated DSB repair, it is unsurprising that loss of BOD1L1 or SETD1A sensitized cells to ionizing radiation and compromised NHEJ. Mechanistically, SETD1A, RIF1, and BOD1L1 act together to prevent resection of DSBs in G1 mediated by BRCA1 together with the nucleases CtIP and MRE11 by promoting H3K4 methylation. A detailed investigation revealed that methylated H3K4 at sites of DNA damage is bound by the N-terminal HEAT repeats of RIF1, stabilizing its recruitment to DSBs [4]. RIF1, together with the Shieldin complex, then blocks DSB end resection and promotes NHEJ [59] (Figure 2C).

Interestingly, this role of BOD1L1/SETD1A may also affect cancer treatment efficacy. Clinically, inhibitors of the enzyme poly(ADP-ribose) polymerase (PARP) have been used with great success in patients with *BRCA*-deficient tumors. However, resistance typically occurs in many patients, which can arise through the loss of factors that promote NHEJ [60–63]. Importantly, loss of BOD1L1 or SETD1A rendered cells insensitive to PARP inhibition, in line with the fact that they are required to promote NHEJ. In agreement, loss of BOD1L1 also compromised class-switch recombination in murine B cells, a process dependent on NHEJ. However, the clinical role of this pathway in PARP inhibitor resistance remains unknown.

## BOD1L1 functions in mitosis

****Although initial experiments failed to reveal a role for BOD1L1 in mitosis [64], a screen for proteins that display altered phosphorylation status following cellular adaptation to a microtubule depolymerizing agent revealed changes in phosphorylation status on the C-terminus of BOD1L1, suggesting a possible cell line or context dependence [64,65]. Interestingly, these phosphorylated sites are targets of Aurora A kinase. Further experiments revealed that BOD1L1 localizes to the mitotic spindle and some kinetochores from early prometaphase through metaphase [65]. Unfortunately, why BOD1L1 only binds some kinetochores remains unknown, as BOD1L1 requires neither microtubules nor Aurora A/B kinase activity for kinetochore localization.

 To gain further insights into BOD1L1 functions in mitosis, a second screen was performed to discover mitotic interacting proteins of BOD1L1. This experiment revealed interactions with Aurora A kinase, NUMA, the anaphase promoting complex/cyclosome, and the condensin complex, suggesting that BOD1L1 might play a role in chromosome condensation during mitosis [65]. Interactions with SETD1A, RAD50, RIF1, MRE11, and XRCC5/6 were also confirmed [2,4,64]. However, the strongest interactions appeared to be with the mTORC2 complex, which plays critical roles in pro-survival signaling, although further experiments are needed to validate and determine the nature of this interaction.

Despite these potentially important interactions, attention was focused on the relationship between BOD1L1 and the PP2A-B56 phosphatase, of which all three components were detected: the PPP2R1A/B scaffold, PPP2CA catalytic, and the PPP2R5E regulatory subunit (Figure 1C) [65]. Immunoprecipitations/pulldowns confirmed these interactions and showed that BOD1L1 interacts with other regulatory subunits, including PPP2R5C and PPP2R5D [65]. The interaction between BOD1L1 and PP2A-B56 offered a mechanistic explanation for why BOD1L1 is differentially phosphorylated after exposure to microtubule depolymerizing agents, since PP2A-B56 plays an important role in stabilizing k-Mt attachments during mitosis by dephosphorylating kinetochore proteins that bind to microtubules [30,66,67]. Phosphorylation of kinetochore proteins results in a negative charge that repels microtubules. Therefore, modulation of phosphatase activity by BOD1L1 could result in resistance to the microtubule depolymerizing agent by stabilizing k-MT binding.

Given that BOD1 also interacts with PP2A-B56, an obvious prediction was that the N-terminal BOD1-like domain of BOD1L1 mediates interaction with PP2A-B56. Surprisingly, the central and C-terminal regions of BOD1L1 interact with the phosphatase, which is the first function to be ascribed to this region of BOD1L1 [65]. It remains unknown whether it is the same site on the C-terminus of BOD1L1 that binds PP2A-B56 and RIF1, or if they have separate binding sites. To understand how BOD1L1 interacts with the phosphatase, AlphaFold was used, which predicted that C-terminal basic stretches of amino acids on BOD1L1 interact with a conserved acidic patch on the regulatory subunits of PP2A [65]. Critically, this acidic patch is present in all five regulatory subunits and is required for dephosphorylation of a subset of PP2A substrates that rely on a basic pH patch in addition to a canonical LxxIxE motif for binding to PP2A. Despite the lack of a defined binding conformation, binding between acidic and basic patches adds substantially to the binding affinity between the phosphatase regulatory subunit and its substrates [68]. Thus, BOD1L1 might also block phosphatase activity. Consistently, following depletion of BOD1L1, reduced levels of phosphorylation were observed on kinetochore PP2A substrate proteins, leading to reduced k-Mt attachment affinity and mitotic defects [65]. Thus, like BOD1, BOD1L1 also regulates phosphatase activity at the kinetochore, albeit through a different mechanism (Figure 2D). How these two proteins evolved to perform similar functions through different mechanisms is yet to be determined.

**Perspectives**

- The biorientation-defective 1 (BOD1) family of proteins has a diverse, important, and growing set of functions, including regulation of chromosome segregation through PP2A-B56, metabolism through the complex of proteins associated with SET1 (COMPASS), DNA repair/fork progression, gene expression through SETD1, and regulation of neuronal function.
- BOD1 and BOD1-like 1 (BOD1L1) appear to have common roles in mitosis and lipid metabolism, but they act through different mechanisms because BOD1 interacts with the PP2A phosphatase through a phosphorylation site that is not present in BOD1L1, whereas BOD1L1 interacts with PP2A through a basic region in the C-terminus. BOD1L1 seems to have a unique role within the family for regulating double-strand break repair and DNA fork progression.
- Important areas for further work will be to determine how BOD1 functions in a neuronal context, what proteins it binds to and which cellular pathways it participates in, whether it does so via the SETD1B/COMPASS complex, and whether BOD1L1/BOD1L2 also participate in these pathways. It is also essential to structurally determine how BOD1 and BOD1L1 bind to the PP2A phosphatase complex and to investigate the functions of the BOD1L1 C-terminal region. Ultimately, this will help determine how the unique features of each family member (e.g. polyproline motif) contribute to their biology.

## Open Access

This article has been published open access under our Subscribe to Open programme, made possible through the support of our subscribing institutions, learn more here: https://portlandpress.com/pages/open_access_options_and_prices#conditional

## Abbreviations

AP-MS: affinity purification-mass spectrometry
ARPP-19: cAMP-regulated phosphoprotein-19
ASD: autism spectrum disorder
ATM: Ataxia-telangiectasia-mutated
ATR: Ataxia telangiectasia and Rad3 related
BOD: biorientation-defective
BOD1L1: BOD1-like 1
CDK: cyclin dependent kinase
CENP-U: centromere protein U
CHK1: Checkpoint Kinase 1
CNV: copy number variation
COMPASS: complex of proteins associated with SET1
CtIP: CtBP interacting protein
DISC1: disrupted in schizophrenia 1
DSB: double-strand break
ENSA: endosulfine alpha
ESC: embryonic stem cell
FANCD2: Fanconi anemia complementation group D2
GGA3: Golgi-associated gamma adaptin ear-containing ARF binding protein 3
HEAT: Huntingtin, eEF3, PP2A, TOR
HEC1: highly expressed in cancer protein-1
KMN: KNL1/Mis12/Ndc80 complex
KNL1: kinetochore scaffold 1
LxxIxE: leucine-X-X-isoleucine-X-glutamic acid
MCAK: mitotic centromere associated kinesin
MCM: Minichromosome Maintenance Complex Component
MIS12: MIS12 kinetochore complex component
MPS1: monopolar spindle-1
MRE11: Meiotic recombination 11
NCD80: NDC80 kinetochore complex component
NHEJ: non-homologous end joining
NMUR2: neuromedin U receptor 2
NUMA: Nuclear Mitotic Apparatus protein 1
PARP: poly(ADP-ribose) polymerase
PFN1: profilin-1
PLK1: polo like kinase 1
PP2A: protein phosphatase 2A
PPP2R5C: protein phosphatase-2 regulatory subunit C, gamma
PV+: parvalbumin-positive
RIF1: replication timing regulatory factor 1
RPA: replication protein A
RPS3: Ribosomal Protein S3
SGO1: Shugoshin 1
SNF2H: Sucrose Nonfermenting 2 Homolog
XRCC5/6: X-ray repair cross-complementing 5/6
k-Mt: kinetochore-microtubule
